# Coacervated and Freeze-Dried Polysaccharides-Nanoparticle with Efficient Encapsulation of Albendazole for High-Performance Treatment of Monogenean Parasite Infestation in Tilapia Fish

**DOI:** 10.3390/ijms27021001

**Published:** 2026-01-19

**Authors:** Andrés Vicent Cubas Rengifo, Norma Lorena Rivadeneyra Sánchez, Chloé Barbosa Teixeira, Rafael R. M. Madrid, Omar Mertins, Patrick D. Mathews

**Affiliations:** 1 Laboratory of IctioParasitology and Applied Nanotechnology (LIPAN), Institute of Biosciences, Sao Paulo State University, Botucatu 18618-689, Brazil; andres.cubas@unesp.br (A.V.C.R.); n.sanchez@unesp.br (N.L.R.S.); chloe.barbosa@unesp.br (C.B.T.); 2 Laboratory of NanoBioMaterials (LNBM), Department of Biophysics, Paulista Medical School, Federal University of Sao Paulo, Sao Paulo 04023-062, Brazil; rafael.madrid@unifesp.br

**Keywords:** chitosan and alginate molecular interactions, nanotechnology in aquaculture, fish parasites, nanoparticle-sustained drug delivery, palatable bioparticles, oral administration, aquaculture industry

## Abstract

Monogenean parasite infestation in fish leads to economic losses in aquaculture, representing a veterinary challenge and an environmental concern. The common administration procedures of anthelmintics to treat monogeneans in fish have low efficiency and diverse drawbacks. In this study, we produced a nanoparticle using chitosan and alginate, biodegradable and biocompatible polysaccharides, as an oral drug delivery material of albendazole anthelmintic for parasite-infected fingerlings of Nile tilapia. The molecular interaction between the biopolymers was optimized and characterized by titration calorimetry. Freeze-drying of nanoparticles resulted in a fine powder with a particle size in the order of 400 nm. The nanoparticles provided 98% encapsulation of albendazole and sustained delivery with predominantly Fickian diffusion. The palatability of the nanoparticle formulation facilitated the oral administration of albendazole. The treatment of 100% prevalence of monogeneans was effective with a six-day dosage providing a total of 915 mg/kg b.w. of drug, resulting in total parasite clearance after 10 days from the treatment beginning, evidenced by microscopy analysis, and no mortality occurred. Therefore, molecular interactions between biofriendly polyelectrolytes yielded albendazole-carrying nanoparticles for high-efficiency parasite treatment in fish farming.

## 1. Introduction

Nowadays, human and veterinary medicine benefit from a wide range of treatments and medications for most known diseases and infections [1,2,3,4,5]. In the veterinary field, major health concerns are associated with animal farming for protein production, driven by increasing human consumption of meat [6,7,8]. The high demands are leading to the intensification of animal farming and breeding, with the fishing industry representing one of the fastest-growing worldwide protein markets [9].

In this context, intensive fish production necessitates constant and efficient health control to optimize production costs and profits, while adhering to regulatory criteria to ensure safe human consumption [10,11]. In fact, there is a direct relationship between the health of farmed animals and the health of consumers, where the transmission of pathogens can pose a threat to public health [12,13].

Parasite infestations in fish represent an increasing threat to fish farming. Indeed, the global aquaculture production has suffered economic losses estimated to be 1 to 10 billion USD annually due to problems related to outbreaks of parasitic and bacterial diseases [14]. However, the economic impacts are not well documented, and comparing them across species and diseases is difficult [15]. Among helminths that parasitize freshwater fishes, monogeneans present a high risk for finfish aquaculture industries, causing expressive economic losses associated with reduced growth, morbidity, and mortality in fish farming around the world, with reported mortalities of the entire fish stock in several cases [16,17,18,19].

Albendazole is a benzimidazole hydrophobic drug known for its action as an anthelmintic for the treatment of human and animal parasite infections. It acts by preventing the worms from absorbing sugar (glucose), which depletes their energy and ultimately kills them. Albendazole has been administered to fish in therapeutic baths [20], mixed with the commercial ration or pellets [21], or via intragastric gavage [22,23]. The drug was studied as an anthelmintic for parasite infections, such as monogenean [24], acanthocephalans [25], digenean trematode [26], and in a variety of wild or farmed fish, such as rainbow trout, tilapia, and Atlantic salmon [27], tambaqui [28], carp [29], and yellow perch [30].

However, the administration of hydrophobic drugs in the aquatic environment is not straightforward, frequently leading to low drug intake and high losses at the bottom of tanks or aquariums, or retention in the water filter system. The administration of albendazole mixed in the ration or in nutritional additives may impact the palatability, resulting in low intake and water pollution. The administration by gavage is evidently unfeasible on a large scale, resulting in a high-cost treatment. Therapeutic baths generally require the use of solvents for drug dispersion, such as ethanol, methanol, and dimethyl sulfoxide, which cause toxicity, side effects, and high levels of water pollution [31,32,33]. Therefore, the majority of current administration procedures of hydrophobic anthelmintic drugs in fish present moderate to high drawbacks and reduced efficacy, leading to unreliable perspectives of practical application.

The development of human and veterinary medicine has been driven by the great expansion of nanotechnology in recent decades. In approaching molecular sciences to nanoscale manipulation and production of functional materials, nanomedicine has provided new solutions ranging from prevention and diagnosis to treatment of diseases, with highly improved efficiency [34,35,36].

Therefore, the evolution of traditional procedures for disease prevention and treatments in aquaculture can also rely on modern nanotechnology approaches [37,38,39]. In this study, we produced a polysaccharide nanoparticle to optimize the administration of albendazole in monogenean-infected cultured fingerlings of Nile tilapia. The biomacromolecules chitosan and alginate, sourced from the aquatic marine environment, were combined in a coacervated and freeze-dried formulation with high entrapment of albendazole, providing an efficient and effective oral drug delivery material for veterinary application in aquaculture.

## 2. Results and Discussion

### 2.1. Thermodynamics of Nanoparticle Production

**Figure 1:**
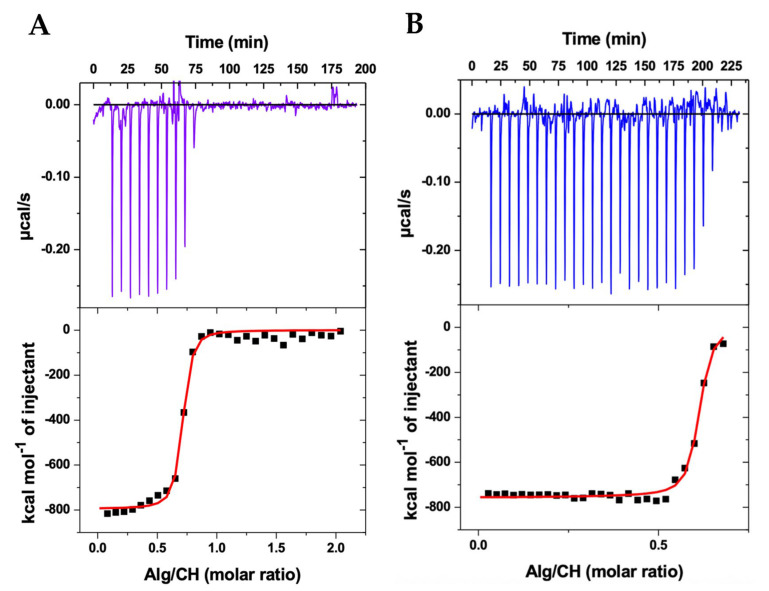
Isothermal titration calorimetry (ITC) results of the thermodynamic interaction between alginate (Alg) and chitosan (CH) during the complex coacervation producing nanoparticles in acetate buffer (pH 4.5) at 25 °C in the presence (**A**) and absence (**B**) of albendazole.

**Figure 2:**
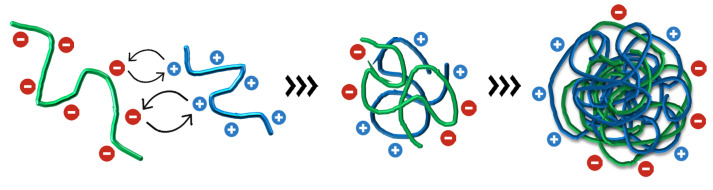
Schematic representation of the electrostatic interaction between negatively charged alginate and positively charged chitosan, which produces a random polyelectrolyte assembly and, after increasing the concentration of the biopolymers, results in the final coiled colloidal nanoparticle with negative and positive surface charges.

Figure 1 shows the results for ITC. The experiment simulated the preparation of the nanoparticles. The upper panels show the heat released (μcal/s) as a function of time at every injection of alginate solution into the cell containing the chitosan solution at the same temperature (25 °C). Every exothermic down-pointing peak represents the energetic variation when alginate comes in contact with chitosan. At every alginate injection, part of the chitosan concentration is “consumed” as the complexation between the oppositely charged polyelectrolytes results in colloidal assemblies, as schematically represented in Figure 2. When the concentration of chitosan in the ITC cell decreased, as it was complexed with incoming alginate, the down-pointing peaks decreased in intensity, and when all chitosan was complexed with alginate, only alginate’s diluting heat variations were recorded, and they were subtracted from the heats of complexation.

The lower panels show the respective integration of the peaks, in kcal/mol of alginate, as a function of the alginate/chitosan molar ratio, thus representing the integrated heats of the alginate–chitosan complexation during the production of the nanoparticles. The integrated heats represent the sum of all energetics involved in the interaction between alginate and chitosan. Since alginate is a polyanion (ionized carboxyl groups) and chitosan is a polycation (protonated amino groups), the electrostatic interaction between the opposite charges (Figure 2) may be considered as the main driving force for the complexation [40,41]. Additionally, hydrophilic and hydrophobic interactions, the breaking of hydrogen bonds, the formation of new hydrogen bonds, van der Waals forces, conformational changes of both biopolymers during interaction, and water molecules release during complexation—all energetics involved in these phenomena may contribute as secondary forces during the polyelectrolytes’ complexation into colloidal nanoparticles [42,43,44]. The sum of all contributions is contained in the integrated heats.

Considering the polyanion and polycation as individual interacting entities, the single set of binding sites model was applied to the integrated heats [40], providing the red sigmoidal curves shown in Figure 1 and thus determining the stoichiometric relation *N* between alginate and chitosan, the equilibrium constant *K*, and the enthalpic variation ΔH of the complexation. The Gibbs free energy was determined as ΔG = −*RT* ln(55.55*K*), where ΔG is defined as the standard state of mole fraction and 55.55 accounts for the concentration of water [45]. The entropic gain was calculated using *T*ΔS = ΔH − ΔG. The results for the complete thermodynamic scenario of the nanoparticle production are shown in Table 1.

As shown in Table 1, the stoichiometric ratio *N* between alginate and chitosan, at the saturation of binding sites, is approximately 0.6 for both nanoparticles containing and not containing the albendazole drug. Hence, around 0.6 alginate monomer per chitosan monomer, or inversely 1/*N*∼1.7 chitosan monomer per alginate monomer at saturation, effectively provides the stoichiometry for the nanoparticle formation at the obtained equilibrium constant *K*. Indeed, the high values obtained for *K* represent the strength of the interaction between the polyelectrolytes, which was slightly higher in the absence of albendazole, although in the same order of magnitude, evidencing a strong tendency of the polyelectrolytes’ assembly and nanoparticles’ formation (Figure 2).

Of notice, despite the lower concentration of alginate solution applied in the ITC experiment in the absence of albendazole, the titration resulted in additional exothermic peaks, as shown in Figure 1B, evidencing that in the end a similar proportion (*N*) between alginate and chitosan is maintained not only when comparing the presence and absence of albendazole, but also when comparing different initial concentrations of the polyanion solution. This outcome further confirms the reproducibility of the nanoparticle production thermodynamics.

The enthalpic variation ΔH obtained in each experiment (Table 1) reflects a strong but similar exothermic process. In addition, almost equal and negative Gibbs energy variations ΔG evidence a thermodynamically favored polyelectrolyte assembly, with a state of lower free energy, thus resulting in the nanoparticle formation, which should therefore be considered as an energetically stable condition [46].

Finally, the entropic contribution *T*ΔS shows almost equal values for CHAlg-Alb and CHAlg, reflecting the condensation of both assembling systems with negative entropy, which may be attributed to the effective electrostatic binding between the oppositely charged biopolymers. Interestingly, other energetics providing entropic increase, e.g., the breaking of hydrogen bonds and water release during the macromolecules’ assembly, were not sufficient to provide a positive entropy, further evidencing that the assembling process is highly energetically favored. These results confirm previous studies on thermodynamic interactions between chitosan and alginate [40,41].

As a matter of fact, the similar thermodynamics on the nanoparticles’ assembly in the presence and absence of albendazole overall evidence that the drug does not prevent interactions between the polyelectrolytes. Hence, the nanoparticles are equally obtained, and the feasibility of producing CHAlg nanoparticles containing albendazole is demonstrated.

### 2.2. Colloidal Features of the Nanoparticles

**Figure 3:**
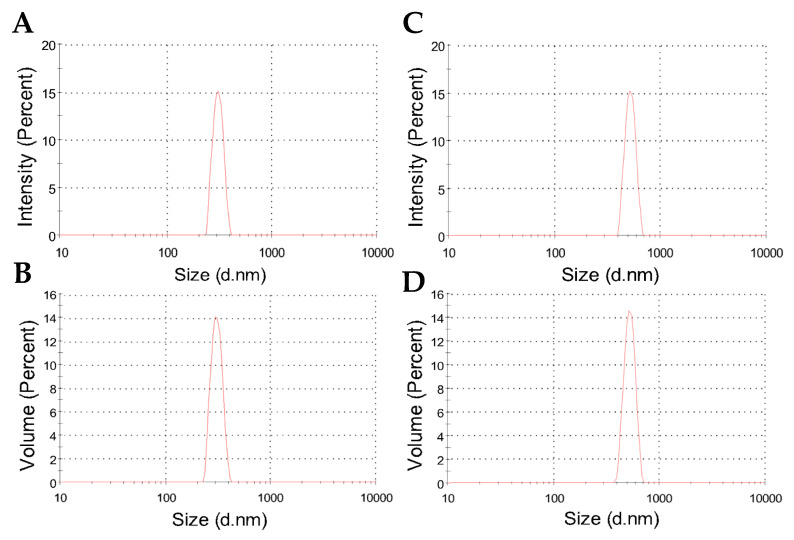
Representative DLS plots showing intensity and volume percents as a function of size distribution for the hydrodynamic diameters of freeze-dried CHAlg (**A**,**B**) and CHAlg-Alb (**C**,**D**) nanoparticles dispersed in water.

Figure 3 shows typical results for the hydrodynamic size distribution of nanoparticles dispersed in water. As evidenced by the curves, a relatively narrow particle size distribution was characterized for both nanoparticles with and without albendazole. Additionally, when comparing the size distribution in terms of intensity and volume percent, the DLS results confirm a consistent and single population of particles.

Table 2 shows the averages of hydrodynamic diameters, which are 382 nm for CHAlg nanoparticles and increase to 475 nm for CHAlg-Alb nanoparticles, indicating that the incorporation of the hydrophobic drug influences the particle colloidal size. Similar trends have been described for nanoparticles containing ivermectin and praziquantel [47,48], where the encapsulation of the hydrophobic drugs led to changes in the fractal dimensionality of the particles as determined by SAXS. In these formulations, it was shown that the colloidal structure is organized as interconnected networks of polymer chains with higher internal distances in the presence of the drugs, resulting in particle expansion. Instead, for “empty” nanoparticles, more compact assemblies fill the internal tridimensional space, providing particles that may have reduced colloidal size. Therefore, in the presence of albendazole, the internal spaces of the polymer network are influenced, thus providing particles of increased hydrodynamic radius. Despite the average size increase in albendazole nanoparticles, the polydispersity for both was in the order of 0.3 (Table 2), confirming that the size distribution was quite similar for particles with and without the drug, as shown in Figure 3.

**Figure 4:**
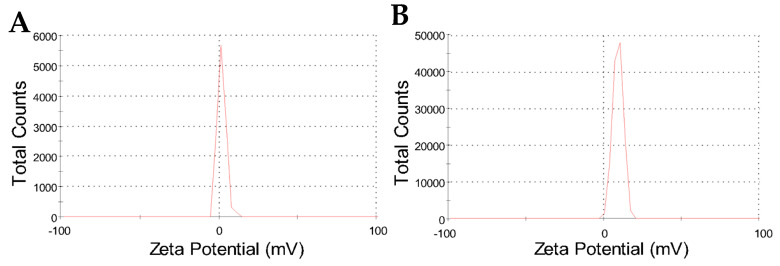
Zeta potential distribution results for CHAlg (**A**) and CHAlg-Alb (**B**) freeze-dried nanoparticles dispersed in water.

However, the zeta potential results have shown a peculiar surface charge profile, comparing CHAlg with CHAlg-Alb. Figure 4 shows the zeta potential results for both nanoparticles, where single and narrow peaks confirm a single population of particles presenting a unique charge profile for each sample. Of note, the peak for CHAlg is centered close to neutral zeta potential at 2.0 mV, whereas the peak of CHAlg-Alb is centered at 9.5 mV (Table 2), despite both nanoparticles being dispersed in aqueous media of similar conductivity.

The zeta potential increase characterized for nanoparticles containing albendazole is not highly significant; however, it indicates, along with the average size increase, that the drug influences, to a certain extent, the colloidal features of the nanoparticles. The slightly positive zeta potential may have important implications for differentiated in vivo performance of the material; for instance, nanoparticles with a positive surface charge may present mucoadhesive and/or muco-penetrating characteristics, as reported for chitosan-*N*-arginine, alginate, and polypeptide nanoparticles in oral drug delivery systems [49].

### 2.3. Scanning Electron Microscopy of Freeze-Dried Nanoparticles

**Figure 5:**
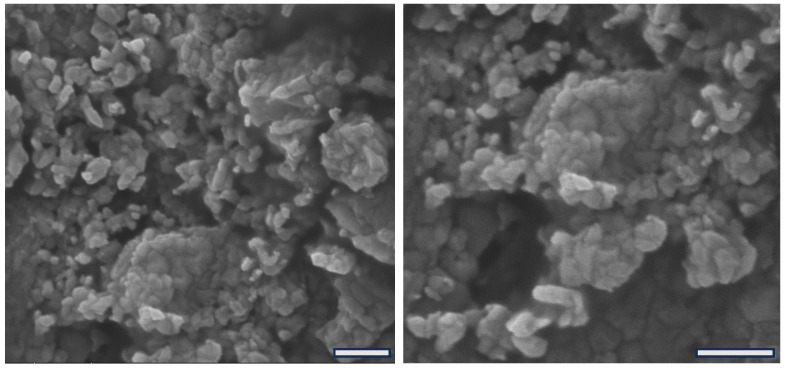
SEM photomicrographs of freeze-dried chitosan–alginate nanoparticles showing two magnifications with scale bars expanding 1 µm.

The process of freeze-dried colloidal nanoparticles can often result in disruption or structural damage of the material, which may compromise its functional activity [50]. We evaluated the structure of the chitosan–alginate nanoparticles by SEM after freeze-drying. Figure 5 shows representative photomicrographs showing the submicrometric and random structure of the nanoparticles. Although clustered by the sample preparation procedure for microscopy observation, it is evident that particles present certain size variation, but all particles are in the nanoscale, i.e., with dimensions under 1 µm.

Furthermore, Figure 5 shows that the nanoparticles present a randomized shape-like structure of various polygonal shapes, unveiling an additional structural feature. However, no microscopic differences were identified between the nanoparticles containing albendazole compared to plain nanoparticles, suggesting that the amount of incorporated drug has not influenced the final microscopic appearance of the particles. For more detailed structural characteristics comparing the particles with and without the drug, the DLS analysis provided improved information as described in the previous section. Moreover, similar to previously reported powder-like nanoparticles [39,49], the macroscopic material produced by freeze-dried nanoparticles affords a fine powder that is easy to handle and easy to administer in the in vivo application.

### 2.4. Encapsulation and Release of Albendazole

**Figure 6:**
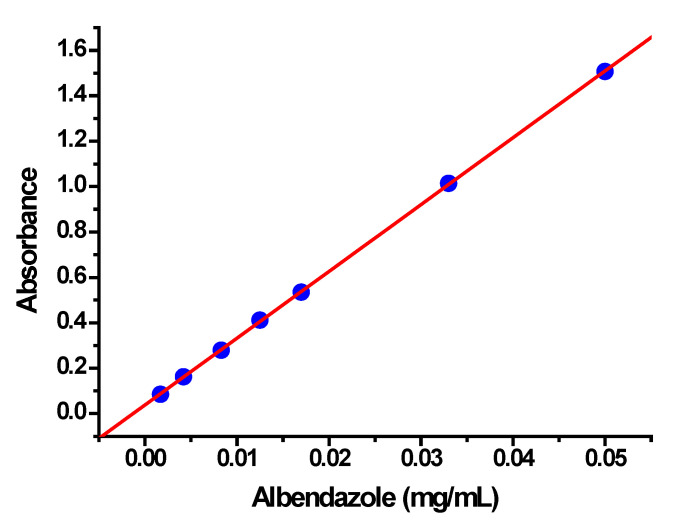
Calibration curve obtained by spectrophotometric measurements of albendazole concentrations in a methanol/water (1:1; *v*:*v*) solution.

The encapsulation and release of albendazole from the freeze-dried nanoparticle formulation were evaluated by centrifugal and dialysis methods, respectively, applying the Lambert–Beer Law to calculate the concentrations. For these aims, an adequate calibration curve was constructed by measuring the absorbance of a range of albendazole concentrations in solution, following previously reported recommendations [51]. Since albendazole is a poor water-soluble drug, a 1:1 (*v*:*v*) methanol/water solution was used in every absorbance measurement. Figure 6 shows the absorbance of albendazole at different concentrations obtained by spectrophotometry.

As shown in Figure 6, the calibration curve presents good linearity with a correlation coefficient (r) of 0.99997, and the slope (B) and intercept (A) of the equation of the regression line are 29.45 and 0.0375, respectively. The Lambert–Beer Law was applied to calculate the unknown concentrations (x), x = (y − A)/B, where y is the absorbance of the centrifuged or dialysis samples in the same 1:1 methanol/water solutions.

The obtained encapsulation percentage of albendazole in the freeze-dried nanoparticles was in the order of 98 ± 1%. The high encapsulation percentage must be reported to the hydrophobic nature of albendazole, which may promote its interaction with the chitosan backbone on the hydrophobic cyclic glucose rings and the hydrocarbon linkages, as well as on the remaining acetyl groups (∼5% of monomers) [52]. The result evidences a successful production of the drug delivery material with an almost complete incorporation of the drug.

**Figure 7:**
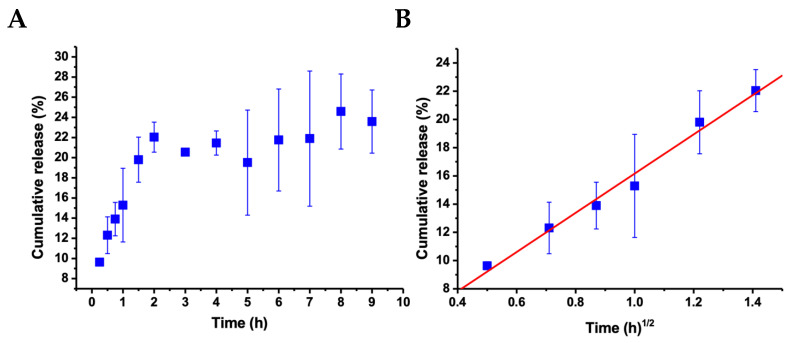
(**A**) Cumulative release of albendazole from CHAlg nanoparticles as a function of time at 25 °C. (**B**) Plot of the same results for the two initial hours as a function of the square root of time, according to the Higuchi model for the release kinetics, showing the corresponding linear regression. Time zero corresponds to the introduction of the sample in the release media, and error bars represent the standard deviation from the average of three independent replicates.

Concerning the release process of albendazole, Figure 7A shows the cumulative release profile as a function of time. As shown, a constantly increasing release was observed during the first two hours, attaining an average of 22% release, after which no considerable increment was observed. To further analyze the release process, different approaches were applied to the data to support the mechanistic and kinetic aspects of albendazole release from the nanoparticles. The best fitting (Figure 7B) was obtained in applying the Higuchi model [53,54], where the increasing linear correlation during the first two hours as a function of the root square of time provides a correlation coefficient r = 0.99287, which was the best fitting compared to the zero-order and Korsmeyer–Peppas models, where the coefficients were lower. The Higuchi model is supported by Fick’s law, considering that the drug release occurs via diffusion from an area of high concentration to an area of low concentration, and the cumulative released amount is proportional to the square root of time [54]. The rate of this diffusion is driven by the concentration gradient and the diffusion coefficient of the diffusing substance, thus describing the movement of the substance through a porous material, which here is the nanoparticle, and then through the solution [55]. The slope of the resulting linear plot in Figure 7B is B = 13.90 ± 0.83, which, for the Higuchi model, represents the release rate constant *K_H_*. The cumulative release of albendazole at around 22% of the encapsulated concentration during the first two hours, followed by the maintenance of this concentration after two hours, can be considered as sustained and controlled by Fickian diffusion.

### 2.5. In Vivo Application and Parasite Treatment

**Figure 8:**
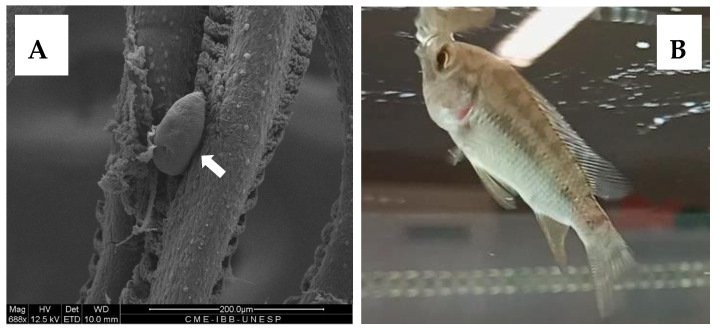
(**A**) SEM Photomicrograph of a monogenean parasite (white arrow) on the gills of tilapia fish with 100% prevalence. (**B**) Fingerling of tilapia fish ingesting freeze-dried powder of albendazole nanoparticles.

The applicability of albendazole nanoparticles was studied in vivo through oral administration to tilapia fish fingerlings. The fish obtained from a local fish farm were thoroughly examined for parasite infection, and monogenean parasites (Figure 8A) were found on the gills of 100% of the fish, with a mean intensity MI = 14.3 ± 2.4 for an initial survey on 10 individuals. The freeze-dried powder of albendazole nanoparticles was provided to the fish by sprinkling it on the aquarium’s water surface, where all the fish immediately swam to eat the powder (Figure 8B). This observation suggests a high palatability of the material, as previously described for polypeptide [49] and chitosan derivative nanoparticles [39,40,41,48].

**Figure 9:**
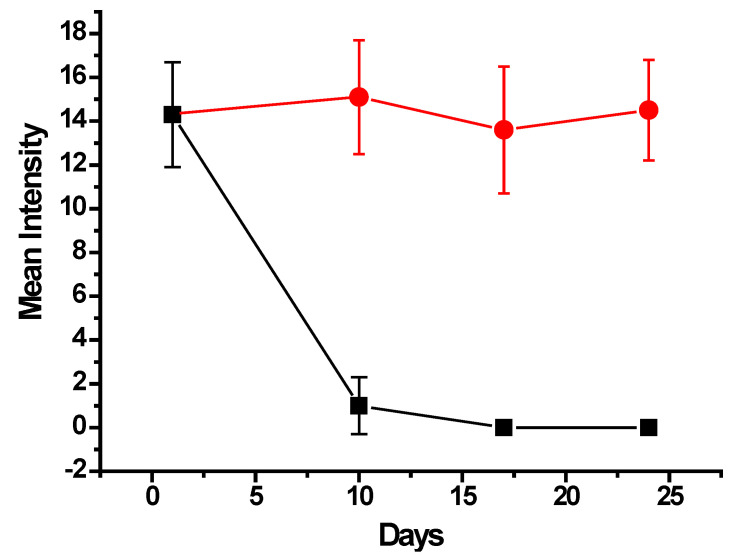
Mean Intensity variation of monogenean parasite infection in tilapia fish for 24 days after the treatment with oral administration of albendazole nanoparticles (black squares) compared to the control of nanoparticles free of drug (red circles).

The parasite treatment was conducted using albendazole nanoparticles, and in parallel, a control experiment was performed in the same conditions, administering nanoparticles without albendazole. As shown in Figure 9, for the control group (red circles), there was almost no variation in the MI of parasite infection during the 24-day experiment. This survey, considering that every day point represented the analysis of 10 fish, amounted to a total of 40 examined fish with MI around 14 parasites per fish with 100% prevalence.

Nevertheless, for the treatment group, the MI significantly decreased to an average of 1.0 ± 1.3 parasites per fish, as shown in Figure 9 (black squares), along with a prevalence of 50%, at 10 days from the beginning of the treatment with albendazole nanoparticles. Then, at the 17th and 24th days, the fish were completely clean, with no parasites found in all examined fish.

The results shown in Figure 9 evidence an effective and efficient treatment of the monogenean parasite infection in fingerlings of farmed tilapia. Considering the concentration of albendazole in the quantity of nanoparticles administered to the total 40 fish, the average total drug dose, which every fish received, was 3.75 mg provided in 6 days in 12 administrations (twice a day). The average weight of all treated fish was 4.10 g, leading to a total administered dose of 915 mg/kg body weight. Alves et al. [20] have described that submitting fingerlings of *Colossoma macropomum* fish in baths containing 500 mg/L provided an efficacy of 48.6% in a 24 h bath. However, in their study, a mortality of 6.6% was reported, and taking into account that albendazole is a hydrophobic drug, an effective application in baths requires the use of solvents such as methanol, ethanol, or DMSO to enable its dispersion in aquarium water. The use of solvents may represent drawbacks in aquaculture, especially concerning toxicity [31,32,33].

In the present study, no mortality and no abnormal behavior were recorded during the whole 24-day period of experiments, neither for the treatment group nor for the control, suggesting that the chitosan–alginate nanoparticles may be considered safe. Moreover, it was evidenced that the nanoparticles possess palatability, which makes oral administration easy and thus enables the actual administration of the carried anthelmintic drug, hence providing the efficacious treatment. As a matter of fact, the nanoparticles containing albendazole represent a promising strategy for parasite treatment in farmed fish.

## 3. Materials and Methods

### 3.1. Materials

Chitosan with a medium average molecular weight (130 kDa) and a high deacetylation degree (95%) was from Primex (Iceland). Alginate (200 kDa) and albendazole (methyl-5-(propylthio)-2-benzimidazol carbamate; analytical standard, 98%) were from Sigma (St. Louis, MO, USA). All other reagents were of analytical grade, and water was from a MilliQ Millipore system with a total organic carbon value of <15 ppb and a resistivity of 18 MΩcm.

### 3.2. Nanoparticles Preparation

Nanoparticles were prepared by the complex coacervation method [40]. Initially, chitosan and alginate were separately dissolved in an acetate buffer (40 mM, pH 4.5) by magnetic stirring for 16 and 2 h, respectively. The alginate solution was titrated into the chitosan solution, either containing or not containing 50 mg of albendazole. The preparations were kept under stirring overnight and then immediately frozen at −80 °C for 3 h. The frozen particles were submitted to lyophilization in a Liotop-Liobras equipment (Liobras, Sao Carlos, Brazil) under vacuum for 72 h. The freeze-dried nanoparticles were kept in a fridge at 4 °C until use.

### 3.3. Isothermal Titration Calorimetry (ITC)

To optimize the stoichiometry of nanoparticle preparation and evaluate the thermodynamics of their formation, ITC experiments were performed using a VP-ITC microcalorimeter (MicroCal Inc., Northampton, MA, USA). Before the experiments, chitosan and alginate solutions were submitted to reduced pressure to avoid the interference of bubbles. The working cell of the equipment with 1.442 mL in volume was filled with chitosan solution. One aliquot of 2 μL, followed by 27 aliquots of 10 μL of alginate solution, was injected stepwise with a 400 s interval into the working cell, which was kept under constant stirring at 307 rpm and at 25 °C. The data acquisition and analysis were carried out with Origin 7 software provided by MicroCal. The data fitting was performed applying the single set of identical binding sites model, as previously described [40].

### 3.4. Dynamic Light Scattering and Zeta Potential

Dynamic light scattering (DLS) was used to determine the size distribution and polydispersity of the freeze-dried nanoparticles dispersed in an aqueous medium. The hydrodynamic radius of nanoparticle dispersion was measured in a Malvern Zetasizer 300 ZS (Malvern Instruments, Worcestershire, UK), operating with a 4 mW HeNe laser at a wavelength of 632.8 nm and detection at an angle of 173°. The measurements were performed in a temperature-controlled chamber at 25 °C. The typical autocorrelation functions were acquired using exponential spacing of the correlation time, and data analysis was performed with software provided by Malvern. The polydispersity was obtained with second-order cumulant analysis of the correlation functions, applying the amplitude of the correlation function and the relaxation frequency.

The zeta potential of the nanoparticles was measured using the same equipment, acquiring at least 100 runs per sample at 25 °C. The principle of the measurement is based on the laser Doppler velocimetry, where the electrophoretic mobility is converted to zeta potential by applying the Helmholtz–Smoluchowski relation. In both experiments, the folded capillary cells were employed.

### 3.5. Scanning Electron Microscopy (SEM)

The morphology of the freeze-dried nanoparticles was evaluated by SEM in an Apreo ChemiSEM System (Thermo Fisher Scientific, Chicago, IL, USA). Samples were prepared by slightly sprinkling the fine powder over a double-sided adhesive carbon tape, which was stuck on aluminum stubs. The stubs were placed in a fine coating ion sputter (Leica EM SCD 500, Wetzlar, Germany) and metalized by sputtering with gold. The samples were exposed to an accelerated voltage of 10.0 kV beam strength and thoroughly scanned for particle surface morphology and size distribution.

Infected tissues fixed in 2.5% glutaraldehyde were post-fixed in 1% osmium tetroxide overnight and dehydrated in a graded ethanol series. The samples were dried in a critical point chamber BALZERS CPD 030 (Balzars, MA, USA) using carbon dioxide and were included in an aluminum stub using a double-sided carbon tape and metalized by sputtering with metallic gold. Samples were visualized with a Quanta 200 scanning electron microscope (Thermo Fisher Scientific, Chicago, IL, USA) operating at 12.5 kV at the Electron Microscopy Center of the Institute of Biosciences of Botucatu, UNESP.

### 3.6. Encapsulation and Release of Albendazole

The percentage of encapsulation of albendazole was determined using the centrifugation–spectrophotometry method [53]. A series of albendazole concentrations, from 0.002 to 0.050 mg/mL, was prepared by dissolving the drug in methanol and diluting to the different concentrations in a 1:1 (*v*:*v*) methanol/water solution. The absorbance of each concentration was measured using spectrophotometry in a K37-UVVIS UV-Visible spectrophotometer (Kasvi, Hong Kong, China) at 220 nm, with the solution used as the standard. These measurements were employed to construct the calibration curve of absorbance as a function of concentration for albendazole and determine the linear fit. A known quantity of freeze-dried nanoparticles containing albendazole was dispersed in 10 mL of water by 1 min vortex mixing and then immediately centrifuged at 11,000 rpm for 30 min (CD 20000 centrifuge, Anco, Hong Kong, China). An aliquot of 1 mL of the supernatant was collected, mixed with the same amount of pure methanol, and the absorbance was measured. The encapsulation percentage was calculated by applying the Lambert–Beer law using the calibration fit [56]. The experiment was executed in triplicate for independent nanoparticle preparations.

The release of albendazole over time was determined using the dialysis method [53]. Briefly, the total amount of freeze-dried nanoparticles containing 50 mg of albendazole was dispersed in 50 mL of water in a dialysis membrane (Servapor, 3500 MWCO) previously washed in water. The dialysis was performed at room temperature (23–24 °C) in a beaker with 90 mL of water under constant magnetic stirring (440 rpm). At every time period, an aliquot of 1.5 mL was collected and diluted with the same volume of methanol, and the absorbance was measured. The volume in the beaker was kept constant by adding the same amount of water. The concentrations were calculated using the calibration fit, and the corresponding drug release profile was represented by the cumulative temporal percent amount of drug released calculated from the total amount of albendazole in the particles. The assays were performed in triplicate, and the average and standard deviation were calculated.

### 3.7. In Vivo Experiments

The in vivo experiments were conducted using fingerlings of Nile tilapia (*Oreochromis niloticus*) fish obtained from an aquaculture farm located in the municipality of Pardinho, State of São Paulo, Brazil (23°04′51″ S, 48°22′26″ W). The fish were naturally infected with monogenean parasites in the farming. An aquarium system with a constant circulating and filtered water (dissolved oxygen: 5.68 ± 0.78 mg/mL; ammonium: 0.02 ± 0.01 mg/mL; hardness: 80 ± 6 mg/mL; pH: 6.5 ± 0.2), thermo-standardized at 26 ± 1 °C, was employed in the treatment experiments. Eight aquariums, each 60 L in volume, were acclimatized to receive the fish, and each aquarium contained a population of 10 fish. The fish were housed in the aquariums for 7 days before the experiments and nourished with the fish’s freshwater ration (TetraMin Tetra flakes) twice a day at 9 a.m. and 4 p.m. Four aquariums were designated for the experiment with the freeze-dried nanoparticles containing albendazole and the other four aquariums for the control with nanoparticles without the drug.

The nanoparticles were weighed, and 50 mg of the powder was administered in each aquarium 20 min before the daily feedings for 6 days, resulting in a total of 2.4 g of nanoparticles with albendazole and the same quantity of nanoparticles without albendazole. The water circulation system was turned off during administration and for an additional period of 30 min to avoid the clearance of nanoparticles in the filters. After the 6 days of treatment, fish were normally fed as before. On the 10th day from the beginning of the treatment, 10 fish were randomly collected from the 4 aquariums of administered albendazole nanoparticles, immediately euthanized by neural pithing [57] and examined for parasite infection using an optical microscope under bright-field imaging and a 10× objective. The same process was performed for the control group. This procedure was repeated after one and two weeks to monitor the treatment progress.

The parasitic indices of prevalence (P) and mean intensity (MI) were calculated according to the criteria by Bush et al. [58] with P (%) = N_P_/N_E_ × 100, where N_P_ is the number of fish infected by parasites and N_E_ is the total number of fish examined, and MI = N_sp1_/N_Psp1_, where N_sp1_ is the number of a given class of parasite and N_Psp1_ is the number of fish infected by a given class of parasite.

## 4. Conclusions

The results of this study demonstrated that the coacervation between chitosan and alginate enabled the reproducible formation of nanoparticles in the presence and absence of albendazole, with similar thermodynamic parameters, confirming that the incorporation of the drug does not alter the polyelectrolyte complexation process. The nanoparticles retained their structural integrity after freeze-drying and a high encapsulation efficiency of albendazole was provided. In addition, the initial release profile exhibited a controlled behavior that fitted the Higuchi model, indicating the predominance of a diffusional mechanism during the early stages of release.

In the in vivo assays, the nanoparticles were spontaneously ingested by the fish, and no mortality was recorded in any experimental group. Oral administration of the albendazole-loaded formulation for six days markedly reduced the initial parasite intensity and provided the complete elimination of monogeneans in subsequent samplings, whereas fish in the control group maintained stable infestation levels throughout the experiment. Taken together, these findings demonstrate that chitosan–alginate nanoparticles enable efficient encapsulation and effective oral delivery of albendazole, representing a viable alternative for the treatment of monogenean infestations in farmed tilapia.

## Figures and Tables

**Table 1:** Thermodynamic results characterizing the interaction between alginate (Alg) and chitosan (CH) in the presence and absence of albendazole (Alb) in the production of the colloidal nanoparticles at 25 °C, obtained from ITC results.

Sample	*N*	*K* (10^9^ M^−1^)	ΔH (kcal/mol)	ΔG (kcal/mol)	*T*ΔS (kcal/mol)
CHAlg Alb	0.682 ± 0.005	5.02 ± 1.31	−794 ± 11	–15.6	778
CHAlg	0.601 ± 0.002	7.60 ± 1.22	−756 ± 43	–15.9	740

**Table 2:** Size and surface charge profiles of the produced nanoparticles, in terms of colloidal hydrodynamic diameter with the corresponding polydispersity index (PDI) and zeta potential with the dispersion conductivity.

Nanoparticle	Hydrodynamic Diameter (nm)	PDI	Zeta Potential (mV)	Conductivity (mS/cm)
CHAlg	382 ± 37	0.28	2.0 ± 2.7	0.62
CHAlg-Alb	475 ± 51	0.33	9.5 ± 3.2	0.82

## Data Availability

We declare that our research data are available on reasonable request.
